# Laparoscopic exploration of the common bile duct and removal of dead worm in a patient of cholangitis after endoscopic retrograde cholangiopancreatography failure

**DOI:** 10.4103/0972-9941.58504

**Published:** 2009

**Authors:** Mushtaq Chalkoo, Ibrahim Masoodi, Shabnum Hussain, Shaheena Chalkoo, Omar Farooq

**Affiliations:** Department of Surgery, Government Medical College, SMHS and Associated Hospitals, Srinagar, Jammu and Kashmir, State, India

**Keywords:** Ascariasis, common bile duct, laparoscopy

## Abstract

We describe a dead ascaris-induced extrahepatic bilary obstruction in a young female who presented with acute cholangitis. The dead ascaris was removed by laparoscopic exploration of common bile duct after endoscopic retrograde cholangiopancreatography failure. Patient had an uneventful hospital course after the procedure and was discharged afebrile after 3 days of hospital stay.

## INTRODUCTION

Biliary ascariasis is a rare cause of obstructive jaundice. The traditional treatment involves endoscopic extraction of the worm. We report a rare case of laparoscopic extraction of a dead worm from the biliary tree after an endoscopic extraction failed.

## CASE REPORT

A 35-year-old female presented to our surgical outpatient department with 4 days history of abdominal pain, jaundice and fever. She had no viral prodrome, history of any offending drug intake or any surgical intervention prior to this presentation. Her past history and her family history were uneventful.

**Figure 1:**
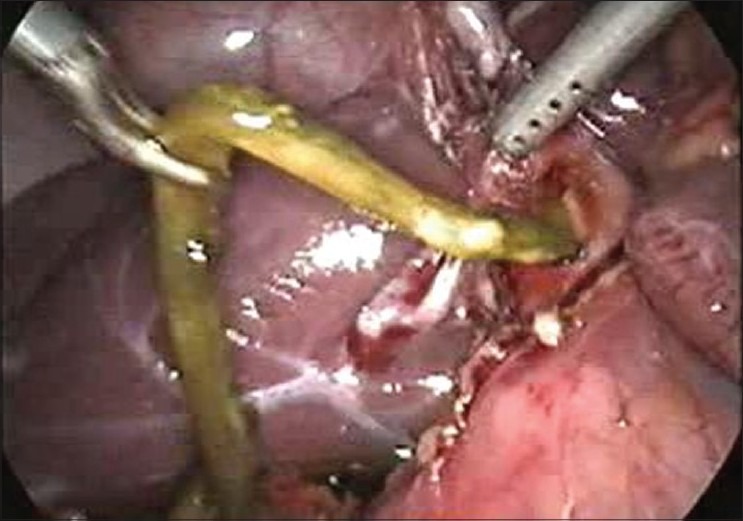
Dead worm being removed from the common bile duct

**Figure 2:**
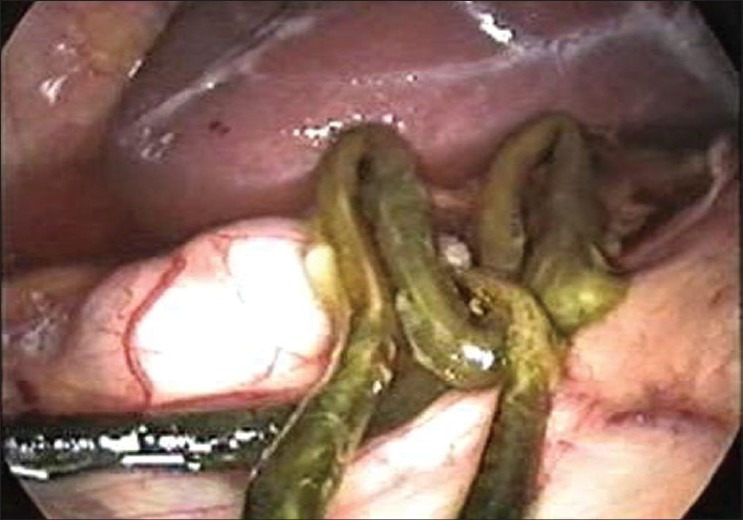
Entire dead worm removed

**Figure 3:**
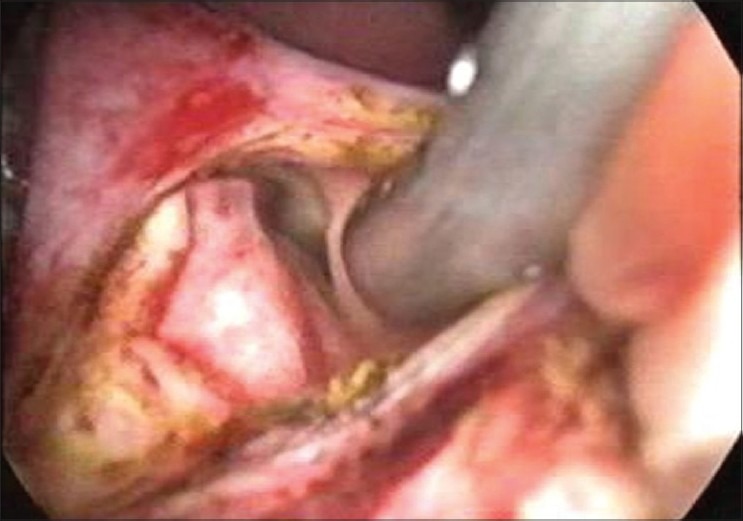
The sucker in the left hepatic duct

On examination, she was conscious, oriented haemodynamically stable and had icterus. She had no oedema or lymphadenopathy or signs of chronic liver disease. She had a pulse rate of 96 beats per minute and body temperature of 101°F. Her abdominal examination revealed soft hepatomegaly 2 cm below the costal margin in the midclavicular line and no splenomegaly or free fluid. Her other systemic examination was normal. On evaluation, she had leucocytosis (TLC 14,500) with predominant neutrophilia (P_82_L_12_). Her liver function test revealed serum bilirubin levels of 6.2 mg/dl and transaminase levels (SGOT and SGPT) of 38 and 42 IU (reference range SGOT/SGPT 22/24). She had marked elevation of serum alkaline phosphatase of 62 KA units (reference range of ALP 11-13 KA units). Her kidney function tests and electrolytes were normal. Her ultrasound revealed an echogenic linear shadow in the common bile duct (CBD) and a grossly dilated intrahepatic biliary ductal system. Her blood cultures were sterile. Her cholangitis wasmanaged with IV fluids and antibiotics, and she was subjected to endoscopic retrograde cholangiopancreatography (ERCP). Despite the multiple attempts made, worm could not be extracted from the CBD. There was some technical problem and we could not visualize the worm through ERCP. She was later taken up for laparoscopic CBD exploration. On laparoscopic examination, the CBD was grossly dilated and a dead worm was removed from CBD [Figures 1 and 2]. We used suction and irrigation in an alternate mode for the removal of the dead worm [Figure 3]. T-tube was placed *in situ*. In the postoperative period, the T-tube was managed the same way as in conventional open choledochotomy. We tried intermittent clamping of the tube from 8^th^ P.O.D and we did not observe abdominal pain or jaundice and on 12^th^ P.O.D a T-tube cholangiogram was done which did not show any filling defects in the CBD. The T-tube was removed uneventfully. She had marked relief in her symptoms and became afebrile. Her bilrubin and serum ALP levels had a progressive fall and she was discharged after 3 days from the hospital afebrile without any complication. She was dewormed and is following our OPD.

## DISCUSSION

Biliary parasitosis is one of the common causes of biliary obstruction in developing countries and can often be confused with stone disease. It is now not limited to the developing countries alone due to increased worldwide travel and immigration.

Endoscopic sphincterotomy and bile ductal clearance, along with pharmacotherapy, are the mainstays of treatment. The success of endoscopic extraction was reported to be in 19/25 by Beckingham *et al*.[1] ERCP failure, however, may be due to the presence of ascariasis in the gallbladder and due to stricture or stones, and these patients require surgical management. After ERCP failure, various options available are surgical exploration and laparoscopic extraction of the living worm and biliary stones as reported by Yoshihara *et al*.[2] These procedures have been found to be very effective for biliary ascariasis with biliary stones after ERCP failure. Astudillo *et al*.[3] recently described successful extraction of parasite by laparoscopy either by CBD exploration or by the combination of choledochotomy and cholecystectomy in a series of 13 cases with biliary ascariasis over a period of 15 years. Can emergency laparoscopic CBD exploration be a better option in failed ERCP than open surgery? This issue was addressed by Gholipour *et al*.[4] who compared emergency laparoscopy to surgery in patients with cholangitis intervened within 72 h of illness in a university tertiary-care hospital. We observed that although operating time was more in laparoscopic surgery, but the average ICU stay, total hospital stay and hence expenditure were significantly less in the laparoscopy group. Emphasizing the expertise in laparoscopic procedures, least complications were observed in the laparoscopic group of patients. Moirangthem *et al*.[5] also reported laparoscopic cholecystectomy and exploration of the bile duct for the removal of round worm with an uneventful postoperative course like that of our patient. Limited data are available on CBD exploration for removal of dead worms. Our patient became afebrile after the procedure, and repeat ultrasound revealed no worms in CBD and no dilatation of intrahepatic ducts. Multiple worms in CBD are well known, and ultrasound is considered a very useful modality for diagnosis and for follow-up. Also, there can be worms in bile ductules that may lead to the persistence of symptoms despite of CBD clearance. Follow-up ultrasound is thus mandatory if symptoms persist. The role of follow-up ultrasound in biliary ascariasis was described by Al absi *et al*.[6] We observed that the characteristic appearance of *Ascaris lumbricoides* was as single or multiple echogenic non- shadowing linear or curved strips with or without echoic tubular central lines that represent the digestive tracts of the worm.

CBD injury is the feared complications of laparoscopic surgery; however, the rate of complication is minimal in expert hands. Other method of management of biliary ascariasis in failed ERCP has been described by Ozcan *et al*.[7] In this study, a percutaneous transhepatic approach with balloon dilatation of the ampulla of Vater and subsequent advancement of roundworms into the duodenum with an embolectomy balloon was used. The procedure was successful, with no major complications. However, such procedures are usually challenging and require advanced equipment and high technical expertise. With advancement and more familiarity in laparoscopic surgeries, we advocate laparoscopic removal of dead worms in biliary ascariasis in failed ERCP cases.

## References

[1] Beckingham IJ, Cullis SN, Krige JE, Bornman PC, Terblanche J (1998). Management of hepatobiliary and pancreatic Ascaris infestation in adults after failed medical treatment. Br J Surg.

[2] Yoshihara S, Toyoki Y, Takahashi O, Sasaki M (2000). Laparoscopic treatment for biliary ascariasis. Surg Laparosc Endosc Percutan Tech.

[3] Astudillo JA, Sporn E, Serrano B, Astudillo R (2008). Ascariasis in the hepatobiliary system: Laparoscopic management. J Am Coll Surg.

[4] Gholipour C, Shalchi RA, Abassi M (2007). Efficacy and safety of early laparoscopic common bile duct exploration as primary procedure in acute cholangitis caused by common bile duct stones. J Laparoendosc Adv Surg Tech A.

[5] Moirangthem GS, Singh CA, Lokendra K, Singh LD (2006). Laparoscopic common bile duct exploration for extraction of a round worm. Trop Gastroenterol.

[6] Al Absi M, Qais AM, Al Katta M, Gafour M, Al-Wadan AH (2007). Biliary ascariasis: The value of ultrasound in the diagnosis and management. Ann Saudi Med.

[7] Ozcan N, Erdogan N, Kucuk C, Ok E (2003). Biliary ascariasis: Percutaneous transhepatic management. J Vasc Interv Radiol.

